# ERCP combined with colonoscopy in the treatment of biliary-colonic fistula: case report and literature review

**DOI:** 10.1093/gastro/goad001

**Published:** 2023-01-25

**Authors:** Hongfei He, Tingting Yu, Yaoting Li, Senlin Hou, Lichao Zhang

**Affiliations:** Biliopancreatic Endoscopic Surgery Department, The Second Hospital of Hebei Medical University, Shijiazhuang, Hebei, P. R. China; Biliopancreatic Endoscopic Surgery Department, The Second Hospital of Hebei Medical University, Shijiazhuang, Hebei, P. R. China; Biliopancreatic Endoscopic Surgery Department, The Second Hospital of Hebei Medical University, Shijiazhuang, Hebei, P. R. China; Biliopancreatic Endoscopic Surgery Department, The Second Hospital of Hebei Medical University, Shijiazhuang, Hebei, P. R. China; Biliopancreatic Endoscopic Surgery Department, The Second Hospital of Hebei Medical University, Shijiazhuang, Hebei, P. R. China

## Introduction

Internal biliary fistula is a potentially serious complication of laparoscopic cholecystectomy [1]. Biliary-colonic fistula is a rare type of internal biliary fistula. Formation of a biliary-colonic fistula can occur as the result of surgical injury, stone obstruction, and biliary hypertension. More infrequent causes include intestinal ulcer and malignant tumor. The formation of a fistula also has the effect of infection and inflammation. While very few cases of biliary-colonic fistulas have been reported, these cases typically present with the aforementioned clinical presentations [2, 3]. In the present case, a biliary-colonic fistula resulting from bile-duct injury after laparoscopic cholecystectomy was identified by endoscopic retrograde cholangiopancreatography (ERCP). We innovatively adopted ERCP combined with colonoscopy to clamp the sinus tract for the patient.

## Case report

**Figure 1.**
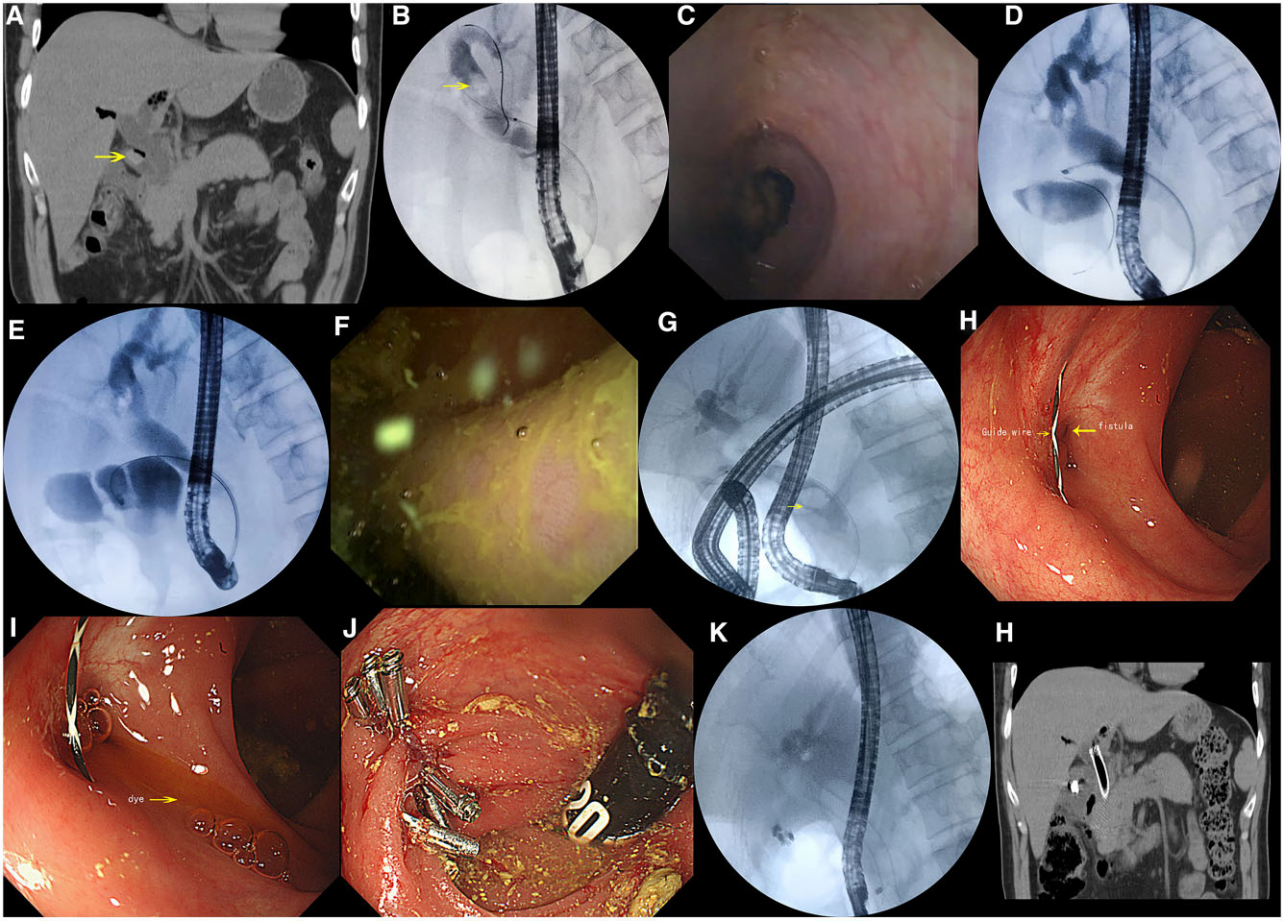
Clinical data of the patient with biliary-colonic fistula. (A) Abdominal computed tomography (CT) showed residual gallbladder neck-duct stone (yellow arrow). (B) Endoscopic retrograde cholangiopancreatography (ERCP) identified choledocholithiasis (yellow arrow). (C) Gallbladder neck-duct stone as seen using the Choledochoscope System. (D) Fluoroscopic image of the guide wire entering the intestinal cavity. (E) The Choledochoscope System enters the intestinal cavity through the sinus tract. (F) Colonic mucosa as seen using the Choledochoscope System. (G) In the second operation, co-use of duodenoscopy and colonoscopy was used. ERCP showed the reflux substance in the bile duct as the guide wire entered the cavity. (H) Guide wire and fistula of colonic hepatic flexure as seen under colonoscopy. (I) Dye injected into the bile duct flowing out from the colonic fistula. (J) Titanium clips were used to clamp the colonic fistula. (K) Fluoroscopic image of the placed biliary stent after recleaning the bile duct. (L) CT examination to confirm biliary stent position.

A 46-year-old male patient was admitted to our hospital with poor appetite and fever for 5 months; the patient had had cholecystectomy due to gallbladder polyps 10 years ago. Physical examination revealed right-upper-quadrant tenderness, soft abdomen, and no jaundice. Abdominal computed tomography (CT) showed a large number of stones in the common bile duct and cystic duct (Figure 1A). We conducted ERCP and used the eyeMax Choledochoscope System Digital Controller (MICRO-TECH, Nanjing, China) to explore the bile duct (Figure 1B and C). After removal of common bile-duct stones, we crossed the guide wire over the cystic-duct stone under the guidance of the Choledochoscope System and used the basket to remove the stone. However, fluoroscopy showed an unusual shadow of the intestine (Figure 1D), which attracted our attention. We explored along the cystic duct again used the Choledochoscope System and identified a sinus tract. Clear colonic mucosa of the intestine could be seen after moving forward (Figure 1E and F). We concluded that the patient's bile-duct stones were caused by reflux due to the formation of a sinus tract between the residual part of the cystic duct and the colon. The sinus tract was likely caused by the cholecystectomy trauma 10 years ago or the repeated friction of the residual cystic-duct stones. After discussion, the surgical team decided to treat it using endoscopy. In the second operation (Figure 1G), the duodenoscope guide wire entered the intestine via the cystic duct and the colonoscope entered through the hepatic flexure of the colon. The guide wire, colon fistula, and dye outflow from the colon fistula were all observed (Figure 1H and I). Eight titanium clips were used to clamp the colon fistula, which blocked the outflow of dye after reinjection (Figure 1J). A 10-mm-diameter and 8-cm-long fully covered self-expandable metal stent (SEMS) was placed through the duodenoscope to compress the cystic duct after the bile duct was cleared with a balloon (Figure 1K). The patient had no post-operative complications and CT reexamination confirmed proper stent positioning (Figure 1L), and the patient’s clinical condition was stable without symptoms at a 3-month follow-up.

## Discussion

Internal biliary fistula refers to the formation of a fistula between the gallbladder or biliary tract and the surrounding organs, which is common in the duodenum, colon, stomach, and liver. Bile flows into the surrounding organs through the fistula and can form a biliary-colonic fistula—an extremely rare type of internal biliary fistula [4]. Sunil Dacha *et al.* [5] reported that one case of gallbladder neck-colonic fistula was found during ERCP; the fistula was caused by the cholecystectomy and colon-repair surgery. A fully covered SEMS was successfully placed and improved the patient's chronic diarrhea. In a separate case, an incorrect surgical clip was placed during the operation that led to a biliary-colonic fistula as identified by ERCP [6]; the patient accepted laparotomy after a plastic biliary stent was placed for decompression. Francisco Igor B. Macedo *et al.* [7] reported on one patient who had undergone laparoscopic cholecystectomy, and magnetic resonance cholangiopancreatography (MRCP) identified a fistula between the biliary tree and intestinal canal; this patient required biliary reconstruction. Justin Smyth *et al.* [8] reported one case of biliary-colonic fistula found by barium enema examination. Because of the patient's poor physical condition and lack of jaundice, the patient was unable to receive ERCP treatment before expiring due to respiratory and renal failure. Many of these patients develop biliary symptoms including jaundice and can suffer from systemic infection because of the existence of abnormal channels between the biliary tract and the intestinal tract, and some patients have chronic diarrhea due to colon fistulas [5]. When diagnosing patients with biliary-colonic fistulas, clinicians should rely on ERCP primarily and avoid abdominal CT, which can be inaccurate. In certain cases, MRCP can be used to identify the injury site.

The key methods used to treat a biliary-colonic fistula are biliary decompression and fistula closure. In the last decade, management of biliary fistulas following laparoscopic cholecystectomy has transitioned to endoscopic therapy with a success rate of between 66% and 100% as reported in the literature. ERCP combined with endoscopic sphincterotomy with stent has become the optimal treatment protocol [1]. The majority of biliary fistulas occur at the cystic-duct stub or a sub-vesical duct of Luschka, which can allow biliary drainage and fistula closure to be considered simultaneously when setting a fully covered metal stent. However, the situation of a biliary-colonic fistula is more complex. In the past, biliary-tract reconstruction surgery was more common than ERCP alone [3, 6]. We present a new combined endoscopic application technique to treat biliary-colonic fistulas in a minimally invasive manner, thus avoiding the second surgical operation. Because of the rarity of these cases, additional studies and lengthier follow-ups are needed to verify the advantage of this technique.

## Conclusions

Faced with a biliary-colonic fistula or other complex biliary and pancreatic diseases, ERCP can become the primary choice for diagnosis and biliary-tract decompression. Compared with surgery, ERCP has fewer complications. With the generalization of minimally invasive concepts and the development of corresponding endoscopic technology, the combined application of different endoscopes can solve more complex diseases. Clinicians should make the best choice after evaluating patients' physical condition, surgical indications, and severity of disease.

## Supplementary Material

goad001_Supplementary_Data

## References

[1] Di Lascia A, Tartaglia N, Fersini A et al Endoscopy for treating minor post-cholecystectomy biliary fistula: a review of the literature. Ann Ital Chir 2018;89:270–7. 30588923

[2] Abiad F, Sidani M. Biliary-colonic fistula through a cystic duct stump. Int Surg 2000;85:231–3. 11325001

[3] Asbun HJ, Rossi RL, Lowell JA et al Bile duct injury during laparoscopic cholecystectomy: mechanism of injury, prevention, and management. World J Surg 1993;17:547–51. 10.1007/BF01655122. 8362534

[4] Verhoeven Y, Bac DJ. Cholecystocolonic fistula. Clin Gastroenterol Hepatol 2011;9:A30. 10.1016/j.cgh.2011.04.014. 21554989

[5] Dacha S, Dwadasi S, Keilin S. Choledochocolonic fistula as the cause for chronic diarrhea. Clin Gastroenterol Hepatol 2017;15:A49. 10.1016/j.cgh.2016.08.040. 27613259

[6] Hanna A, Aneese AM, Cappell MS. Biliary-colonic fistula associated with high-grade biliary stenosis from errant surgical clip during previous biliary surgery: diagnosis and treatment by ERCP. ACG Case Rep J 2021;8:e00617. 10.14309/crj.0000000000000617. 34124279 PMC8189637

[7] Macedo FI, Casillas VJ, Davis JS et al Biliary-colonic fistula caused by cholecystectomy bile duct injury. Hepatobiliary Pancreat Dis Int 2013;12:443–5. 23924505 10.1016/s1499-3872(13)60070-3

[8] Smyth J, Dasari BV, Hannon R. Biliary-colonic fistula. Clin Gastroenterol Hepatol 2011;9:A26. 10.1016/j.cgh.2011.04.015. 21600303

